# Robo-Lector – a novel platform for automated high-throughput cultivations in microtiter plates with high information content

**DOI:** 10.1186/1475-2859-8-42

**Published:** 2009-08-01

**Authors:** Robert Huber, Daniel Ritter, Till Hering, Anne-Kathrin Hillmer, Frank Kensy, Carsten Müller, Le Wang, Jochen Büchs

**Affiliations:** 1 AVT. Biochemical Engineering, RWTH Aachen University, Worringerweg 1, 52074 Aachen, Germany; 2 m2p-labs GmbH, Forckenbecksttr. 6, 52074 Aachen, Germany

## Abstract

**Background:**

In industry and academic research, there is an increasing demand for flexible automated microfermentation platforms with advanced sensing technology. However, up to now, conventional platforms cannot generate continuous data in high-throughput cultivations, in particular for monitoring biomass and fluorescent proteins. Furthermore, microfermentation platforms are needed that can easily combine cost-effective, disposable microbioreactors with downstream processing and analytical assays.

**Results:**

To meet this demand, a novel automated microfermentation platform consisting of a BioLector and a liquid-handling robot (Robo-Lector) was sucessfully built and tested. The BioLector provides a cultivation system that is able to permanently monitor microbial growth and the fluorescence of reporter proteins under defined conditions in microtiter plates. Three examplary methods were programed on the Robo-Lector platform to study in detail high-throughput cultivation processes and especially recombinant protein expression. The host/vector system *E. coli *BL21(DE3) pRhotHi-2-EcFbFP, expressing the fluorescence protein EcFbFP, was hereby investigated. With the method 'induction profiling' it was possible to conduct 96 different induction experiments (varying inducer concentrations from 0 to 1.5 mM IPTG at 8 different induction times) simultaneously in an automated way. The method 'biomass-specific induction' allowed to automatically induce cultures with different growth kinetics in a microtiter plate at the same biomass concentration, which resulted in a relative standard deviation of the EcFbFP production of only ± 7%. The third method 'biomass-specific replication' enabled to generate equal initial biomass concentrations in main cultures from precultures with different growth kinetics. This was realized by automatically transferring an appropiate inoculum volume from the different preculture microtiter wells to respective wells of the main culture plate, where subsequently similar growth kinetics could be obtained.

**Conclusion:**

The Robo-Lector generates extensive kinetic data in high-throughput cultivations, particularly for biomass and fluorescence protein formation. Based on the non-invasive on-line-monitoring signals, actions of the liquid-handling robot can easily be triggered. This interaction between the robot and the BioLector (Robo-Lector) combines high-content data generation with systematic high-throughput experimentation in an automated fashion, offering new possibilities to study biological production systems. The presented platform uses a standard liquid-handling workstation with widespread automation possibilities. Thus, high-throughput cultivations can now be combined with small-scale downstream processing techniques and analytical assays. Ultimately, this novel versatile platform can accelerate and intensify research and development in the field of systems biology as well as modelling and bioprocess optimization.

## Background

Microbioreactors – basically mini-factories for cultivating microorganisms of economic value – have gained increasing acceptance in industry and academic research. Important applications of microbioreactors (MBRs) include screening of medium compositions and clones as well as bioprocess development, optimization and validation [1-4]. The biggest area where MBRs are applied is in the field of structural genomics [5] and screening processes for new biocatalysts such as enzymes [6]. Microtiter plates are predominantly used in such bioprocesses, and these mostly consist of multiple cultivation steps (e.g. generation of cryocultures after clone picking, precultivation, main cultivation with recombinant protein expression) [5,7]. Only little research is being conducted to investigate such processes systematically and in more detail regarding growth kinetics of different clones in multiple cultivation steps. Nevertheless, different growth kinetics can have tremendous effects on clone selection in high-throughput cultivations [8]. Monitoring growth kinetics is a prerequisite for understanding the whole process, for example, for choosing the right time for inoculation from a preculture, for inducing protein expression at an optimal time and, for determining the best harvesting point (e.g. to avoid proteolytic degradation in the stationary phase).

So far it has been difficult to study such complicated, highly parallel processes because of a lack of monitoring tools and sensors for small-scale culture vessels (black-box operation) [4]. Conventional approaches (e.g. off-line analysis of bacterial growth in parallel reactors) used today are too time-consuming. In addition, it is also very tedious to investigate in detail recombinant protein production in small-scale cultivations, e.g. in shake flasks. The induction point for protein expression in *E.coli *as well as the inducer concentration are the most critical factors which influence product yield [9,10]. Interestingly, only few studies have systematically investigated this aspect [9]. These studies entail exhaustive work (e.g. induction at different times, drawing samples, optical density (OD) measurement with dilution of samples, protein analytics via SDS-PAGE or other assays). This might be the reason, why in many laboratories a generic procedure is applied for routine protein expression, namely to induce cells in the early to mid-log growth phase with an IPTG (Isopropyl-beta-D-thiogalactopyranoside) 'standard concentration' of 1 mM [7,9]. However, protein expression is also influenced by many other factors, such as the host/vector system, promoter stength, characteristics of the target protein, media composition and culture conditions [9,11]. Therefore, a generic approach may often lead to suboptimal induction conditions and is normally unsuitable for taking into account multiple parameters to understand and optimize specific expression systems. A MBR system that automatically checks at least the influence of the two main parameters – induction time and inducer concentration – would help to systematically investigate and understand a given host/vector system and could contribute to better expression results than the generic procedure.

Induction at different growth phases leads to great variations in product formation [9]. Therefore, when different clones of a clone library have to be compared regarding recombinant protein yield, it is necessary to induce the clones at comparable biomass concentrations. The conventional clone screening approach would be to monitor the growth of a limited number of clones off-line (e.g. via OD measurement) and then induce them at an appropiate time. This, however, is tedious and not feasible for a large number of clones. Studier considers it even impossible to monitor growth and induce parallel cultures at the same growth phase in high-throughput cultivations [12]. Consequently, the autoinduction medium has been developed. Being a highly sophisticated method, it requires no manual addition of IPTG. It automatically induces the cultures with lactose after the cells have consumed a certain amount of glucose [12]. Nonetheless, this principle cannot be applied for all host/vector systems. Therefore, it would be useful to have a system that permanently monitors growth of different clones in parallel MBRs and uses this information to automatically add inducer at the same physiological state of the cultures.

MBRs have also become increasingly important for industrial production processes. Here, many microbial processes are still poorly unterstood, especially in the area of recombinant protein expression [13]. Hence, process analytical technology (PAT)-driven initiatives tend to use scale-down systems such as (disposable) MBRs in process characterization and validation [4,14]. In these areas, there is also a strong need to combine different unit operations (upstream, downstream and product analysis) in small-scale automated routines [3,15], which demand automation, high-throughput sampling and, indeally, direct product analysis from MBRs [4]. Quantitative kinetic data for protein production are also needed in systems biology to validate dynamic mathematical models of the regulatory mechanisms and networks of expression systems. Therefore, the use of parallel MBRs with integrated on-line-monitoring of product formation would be highly beneficial in systems biology applications and, in general, for industries producing recombinant proteins.

To summarize, the development and application of more advanced MBRs, sampling and measurement devices is necessary for better understanding biological systems and whole bioprocesses [16]. Consequently, as demonstrated above, many disciplines of biotechnological research and development that use MBRs require:

1) MBRs with advanced sensing technologies capable of providing high-content data of key cultivation parameters (pH, dissolved oxygen tension DOT, biomass) and especially protein production;

2) flexible, automated cultivation systems to reduce human manual error and work load, increase throughput and enable the investigation of complex workflows (e.g. multiple cultivation steps); and

3) modular platforms for allowing the combination of easy-to-use, disposable MBRs with downstream processes and analytical assays.

Various systems that partially fulfill these requirements will be discussed as follows. Only systems which use MBRs with volumes of approximately 0.1 to 10 mL (excluding microfluidic devices) will be presented.

Many devices for high-troughput cultivation of microorganisms on a millilitre scale evolved in the last years [3,17-20] and some of them have already been commercialized (e.g. systems from Applikon, BioProcessors, Fluorometrix). MBR automation has been adressed in some systems. There are two main concepts to implement automated cultivation systems. The first one is to integrate MBRs in existing liquid-handling workstations or to combine them with standard robotic devices. For instance, Puskeiler et al. have developed such a system, consisting of a MBR device (called bioreactor block) integrated in a liquid-handling robot [20]. Zimmermann and Rieth established a system that utilizes microtiter plates and standard robotic equipment in a climate chamber to screen mutant libraries [21]. Another MBR device with 24 individual minireactors (M24, Applikon) can be combined with a plate crane and a single-channel pipettor for sampling and feeding [14].

The second concept to automate the operation of MBRs is to design and build completely new combinations of devices for a particular task. Such systems are highly sophisticated and consist of multiple devices (MBR, robotic arm, sampling module, sensing module), which are linked to each other via complex control software. In order to provide a fully automated option for protein expression and purification in high-troughput, a special cultivation system with discontinuous optical density measurement (Piccolo™) has been developed for *E.coli *or insect cell expression systems [22]. Another automated system (SimCell™) uses cell culture MBR arrays that are handled in a cluster design around a central robotic arm [23]. Because such systems are highly complex and need special equipment and periphery, they tend to have high investment costs, prohibiting the widespread use in industry and even more in academia.

On-line monitoring of pH and DOT is realized in many of these MBR systems [3,4]. However, only few of them are capable of monitoring in detail the most dominant indicators characterizing a biological production system, i.e. the formation of biomass and product. Some of these systems measure microbial growth via optical density at-line (sample is removed from the reactor), although this has the disadvantage of only limited data density [3,20] and sometimes requires interruption of the cultivation process. Furthermore, none of the above described systems have the possibility to conduct fluorescence protein monitoring on-line in a large number of parallel MBRs.

Unlike most MBR systems, the so-called BioLector technique provides a cultivation system that is able to permanently monitor microbial growth, fluorescence of reporter proteins, pH and DOT under defined conditions in microtiter plates without interrupting the shaking movement [24,25]. Using microtiter plates offers the advantage of high-throughput, low costs, standardization and thoroughly studied engineering parameters for cultivation of microorganisms [2,26-30]. Furthermore, microtiter plates – being the international standard for laboratory automation – offer widespread and easy automation possibilities and allow high-throughput bioprocess development [24,31].

This work presents the development and first results of a new concept for an automated microfermentation platform (for shortness here called 'Robo-Lector'). This platform combines a liquid-handling workstation with a BioLector, resulting in a flexible system to study small-scale cultivation processes in detail. Here, the following three automated methods using the Robo-Lector are described:

1) 'induction profiling' comprises a method to investigate the influence of induction time and inducer concentration on protein expression;

2) 'biomass-specific induction' is a method that enables one to induce cultures with different growth kinetics at a similar physiological state (meaning that the different wells are induced at different specific time points); and

3) 'biomass-specific replication' is a method that equalizes the biomass concentration of a preculture microtiter plate for further experiments.

In these methods, a common expression system (*E.coli *BL21(DE3) with a plasmid harboring a fluoresencent reporter protein under the control of the T7 promoter) was used to study in detail recombinant protein expression of this host/vector system and to validate the established platform.

## Methods

### Organism

For all experiments the strain *E. coli *BL21(DE3) pRhotHi-2-EcFbFP was used (kindly provided by T. Drepper, Institute of Molecular Enzyme Technology, Heinrich-Heine-University Düsseldorf, Germany) (Katzke N, Arvani S, Bergmann R, Circolone F, Markert A, Svensson V, Jaeger KE, Heck A, Drepper T: A novel T7 RNA polymerase dependent expression system for high-level protein expression in the phototrophic bacterium *Rhodobacter capsulatus*, submitted). The used expression plasmid harbors the T7 promoter that is under the control of the *lac *operator and a kanamycin resistence gene. The fluorescent protein EcFbFP was used as a model protein. Therefore, the EcFbFP encoding gene was cloned into the pRhotHi-2 vector downstream of the T7 promoter. A His_6_-tag was fused to the C-terminus of the EcFbFP resulting in a recombinant fusion protein with a molecular weight of 16.5 kDa. The gene for this FMN-binding fluorescent protein (FbFP) was codon-optimized for expression in *E.coli *(hence, the name EcFbFP) and could even fluoresce in the absence of oxygen (in contrary to GFP and its derivates) [32]. The used FbFP is now commercially available under the trademark evoglow (evocatal GmbH, Düsseldorf, Germany).

### Medium and Solutions

For all cultivation experiments, MDG mineral medium with glucose as a carbon source was used [12]. It consists of of 25 mM Na_2_HPO_4_, 25 mM KH_2_PO_4_, 50 mM NH_4_Cl, 5 mM Na_2_SO_4_, 2 mM MgSO_4_, 0.2 × trace metals (stock solution consists of 50 mM FeCl_3_, 20 mM CaCl_2_, 10 mM each of MnCl_2 _and ZnSO_4_, and 2 mM each of CoCl_2_, CuCl_2_, NiCl_2_, Na_2_MoO_4_, Na_2_SeO_3 _and H_3_BO_3_), 18.8 mM aspartate and 27.8 mM glucose. Additionally, 50 μg/mL kanamycin was added to the medium. To induce protein expression, sterile filtered IPTG stock solutions of different concentrations (0.2 mM to 30 mM) were applied. All chemicals were of analytical grade and supplied by Carl Roth (Crailsheim, Germany) or Sigma (Taufkirchen, Germany).

### Cultivation

All the cultivations were carried out in sterile black 96 well microtiter plates (μClear, Greiner Bio-One, Frickenhausen, Germany) in the MBR system BioLector (m2p-labs, Aachen, Germany) [25]. The microtiter plates were sealed with sterile pierceable, resealable tape (X-Pierce, Excel Scientific, Victorville, USA), allowing ventilation of the wells at reduced evaporation rates. The following conditions were applied for all cultivations in the BioLector: temperature 37°C, shaking diameter 3 mm, shaking frequency 950 rpm, relative humidity in the incubation chamber 80% (typical evaporation rates were 5 vol.-% per day). The EcFbFP fluorescence was monitored at an excitation of 460 nm and an emission of 492 nm. The biomass concentration was measured via scattered light intensity (I) [24,25] and was detected at an excitation of 620 nm. The initial scattered light intensity (I_0_) was mainly attributed to such factors as the media background or the type of the microtiter plate and was substracted from the residual scattered light data (I-I_0_) [24]. There is a general linear correlation between scattered light and OD [25]. In the current work, the scatterd light data can be correlated to OD values according to the equation OD = scattered light/22.4, which was determined with a calibration curve (similar to [25]). Nevertheless, for comparing different cultures in parallel experiments, a calibration to OD values is not essential. Hence, the biomass data are shown as scattered light intensity in arbitrary units. The measurement cycle for EcFbFP and scattered light monitoring was 5 min for the method 'induction profiling'. This means that the EcFbFP and scattered light signals were measured every 5 min in every single well of a microtiter plate. For the method 'biomass-specific induction' this cycle time was 4 min and for the 'biomass-specific replication' method the scattered light was monitored every 10 min. The total filling volume per well at the beginning of the cultivation was 190 μL. Precultures were made in a 250 mL shake flask under the following conditions unless otherwise stated: inoculation with a cryoculture to yield an OD at the start of 0.1, temperature 37°C, total filling volume 10 mL of MDG mineral medium, shaking diameter 50 mm, shaking frequency 350 rpm, duration 16 to 24 h.

### Design of the automated microfermentation platform

**Figure 1:**
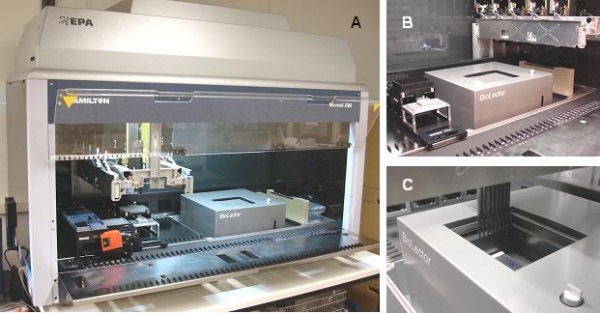
**The Robo-Lector automated microfermentation system**. (A) BioLector (m2p-labs) integrated into a liquid-handling workstation (Microlab STAR, Hamilton Robotics). (B) detail view of the integrated BioLector. (C) 8 channel pipetting head adding liquid to a microtiter plate in the BioLector.

The Robo-Lector system presented in this study was made up of the MBR system BioLector and a liquid-handling workstation (Microlab STAR, Hamilton Robotics, Martinsried, Germany) (Figure 1). Here, a custom-built BioLector was placed down into the free space under the deck of the pipetting robot. The height of the BioLector setting was adjusted so that the traverse height of the pipetting arm did not interfere with the BioLector. Moreover, the liquid-handling workstation was equipped with a HEPA (High Efficiency Particulate Air Filter) hood providing an air stream with a very low particle fraction for transferring and storing liquids (e.g. medium, inducer stock solutions) under sterile conditions. The pipetting robot was connected to a computer and actuated via a control software (Vector software, Hamilton Robotics). In addition, the BioLector was connected to the same computer and controlled with the BioLection software (m2p-labs, Aachen); both devices were connected via a TCP/IP-network. Different command codes for controlling the BioLector (e.g. open lid, pause measurement) were implemented in the Vector control software of the pipetting robot, thereby allowing the integration of the BioLector in complex workflows including liquid-handling processes and microtiter plate movements. Different methods were programed, tested and optimized regarding time schedules and ease of use.

### Induction profiling

**Figure 2:**
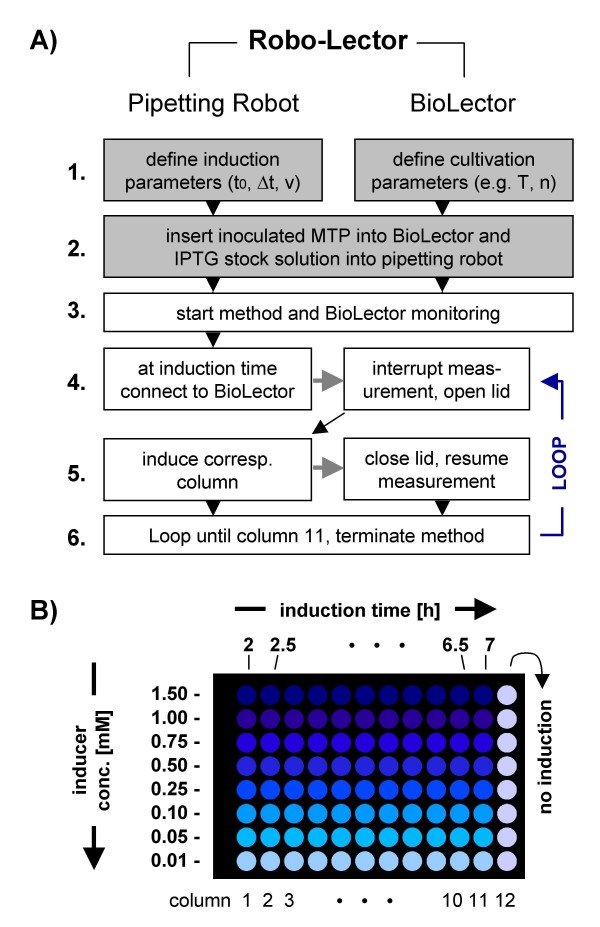
**Principle of method *induction profiling***. (A) Simplified process flowchart; shaded boxes indicate necessary user input; gray arrows indicate interaction of the pipetting robot and the BioLector; MTP: microtiter plate; parameters t_0_: time of first induction, Δt: time interval between two induction points, v: volume of inducer added per well, T: cultivation temperature, n: shaking frequency; please see Materials and Methods section for more details. (B) Established experimental procedure for induction profiling used in this work.

The induction profiling method was established to study recombinant protein expression in host cells that require inducing agents (e.g. IPTG, arabinose) to start protein production. A method was programed for the liquid-handling platform that enables the automatic induction of up to 96 cultures of a recombinant strain in a microtiter plate with varying inducer concentrations at different times. The principle of this so-called induction profiling method is shown as a simplified flowchart in Figure 2. To start the method, the user first has to define the induction parameters in the program, i.e. the first point of induction (t_0_), the time interval between two induction points (Δt) and the desired volume of inducer to be added to each well (v) (Figure 2A, step 1.). Besides that, the cultivation parameters for the BioLector has to be defined (step 1.), e.g. the desired temperature (T) or the shaking frequency (n) (see also *Cultivation *section).

Then, a microtiter plate with medium is inoculated with a preculture of *E. coli *BL21(DE3) pRhotHi-2-EcFbFP (to give 190 μL of culture with an OD of 0.1) and placed inside the BioLector (step 2.). In parallel, a microtiter plate with 8 different IPTG stock solutions ranging from 0.2 mM to 30 mM is placed inside the pipetting robot. At the different times of induction, 10 μL of the 8 stock solutions are transferred to the corresponding wells of the culture plate in the BioLector, thereby yielding IPTG concentrations of 0.01 to 1.5 mM (Figure 2B). The aforementioned steps are manually conducted by the user, whereas after the start of the method (step 3.) the whole process (monitoring of the culivation in the BioLector, addition of inducer) is run completely automated.

Once the cultivation reaches the predefined first induction point (2 h in this study), the liquid-handling robot picks up 8 sterile filtered tips and finally aspirates 10 μL of each IPTG stock solution. Thereafter, the pipetting channels moves over the BioLector lid. Then the pipetting robot software links up to that of the BioLector, interrupts the measurement and opens the BioLector lid (step 4.). When the lid opens, the shaking movement in the BioLector stops, and the IPTG solutions are dispensed into the first column of the culture plate (taking less than 20 seconds). After the induction with IPTG, the lid is closed and the cultivation and measurements resume (step 5.). This induction process is repeated in a loop (step 6.) with the predefined time interval Δt (0.5 h in this study) until column 11 of the culture plate is attained (induction after 7.5 h). Here, column 12 is not induced and serves as a reference. The resulting induction conditions for every culture in a well is shown in Figure 2B.

### Biomass-specific induction

**Figure 3:**
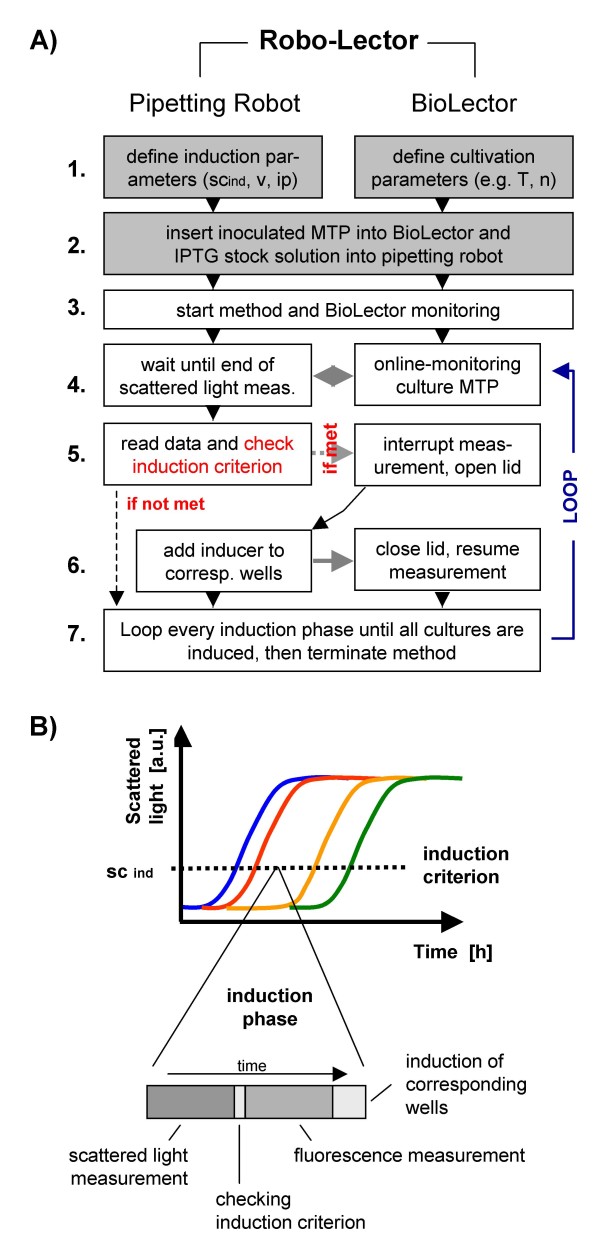
**Principle of method *biomass-specific induction***. (A) Simplified process flow chart; shaded boxes indicate necessary user input; gray arrows indicate interaction of pipetting robot and BioLector; MTP: microtiter plate; parameters sc_ind_: scattered light at induction (induction criterion), v: volume of inducer added per well, ip: induction phase, T: cultivation temperature, n: shaking frequency. (B) Schematic principle of the established procedure for biomass-specific induction; throughout the complete cultivation, subsequent induction phases are conducted for the whole microtiter plate; please see Materials and Methods section for more details.

With this method, the biomass in a main culture is permanently monitored using the BioLector and the cultures are induced at a specific, predefined biomass concentration. The principle of this so-called biomass-specific induction method is shown as a simplified flowchart in Figure 3A. Again, the user first has to specify the cultivation conditions of the BioLector. Moreover, the parameters for the induction step are defined (step 1.). These included:

1) the target biomass concentration, at which all the cultures of a microtiter plate are induced (scattered light at induction sc_ind_, also called induction criterion);

2) the inducer volume added per well v (10 μL); and

3) the induction phase ip (4 min). This specific time interval is defined to avoid that the BioLector is interupted too often for induction, as this would eventually have negative effects on the oxygen supply and as a consequence the performance of the cells.

The whole cultivation process is divided into subsequent induction phases, which are conducted for the whole microtiter plate. During the induction phase, the scattered light and EcFbFP fluorescence are measured whereby the induction criterion is also checked and induction takes place in the corresponding wells which reached the induction criterion (Figure 3B).

After the above parameters for the BioLector and the induction step were set, an inoculated microtiter plate is placed in the BioLector (step 2.). In this model experiment, the wells of the microtiter plate were inoculated to give initial biomass concentrations of OD 0.16, 0.11, 0.05 and 0.03. Respectively, six wells were inoculated with each initial OD concentration (resulting in 24 cultures) to simulate different growth kinetics and to test the feasibility of the method. In addition, a sterile plate with an IPTG stock solution (2 mM) was placed inside the pipetting robot (step 2.). These steps are conducted manually. Afterwards, the method and the BioLector monitoring is initiated (step 3.).

The BioLector monitors the culture microtiter plate and waits until the scattered light measurement is finished for the whole plate (Figure 3A, step 4.; Figure 3B). These data are then read from the BioLector and checked to see if the induction criterion is met (Figure 3A, step 5.; Figure 3B). Meanwhile, the BioLector continues with the monitoring of the EcFbFP fluorescence in each well (Figure 3B). The following program is then run accordingly. If no culture meets the induction criterion, the program waits until the next induction phase (Figure 3A, 'if not met', go from step 5. to step 7.). The other condition is that at least one well reaches a scattered light intensity greater than the induction criterion sc_ind _(50 a.u., OD 2.2) (Figure 3A, 'if met'). Then, the program calculates how much inducer is needed in every one of the 8 rows of the plate (e.g. 3 wells in row 5 have to be induced; this requires 30 μL of IPTG to be aspirated by one pipetting channel). Afterwards, the liquid-handling robot aspirates the appropiate volume from the plate with the IPTG solution with 8 sterile tips and moves to the BioLector lid. The program waits until the product fluorescence measurement is completed (Figure 3B). Subsequently, the robot software then links to the BioLector software, interrupts the measurement and opens the BioLector lid (Figure 3A, step 5.). Once this lid opens, the shaking movement in the BioLector is interrupted for 20 to 30 seconds (depending on the number of wells that have to be induced) and the IPTG solutions are dispensed into the corresponding wells that reached the induction criterion (10 μL per well). After the induction, the lid is closed and the BioLector cultivation and measurement resumes (Figure 3A, step 6.). This workflow of measuring, checking and induction is repeated in a loop at the predefined time of the induction phase (step 7. to 4.). This loop is repeated until every culture of the microtiter plate has been induced.

### Method biomass-specific replication

**Figure 4:**
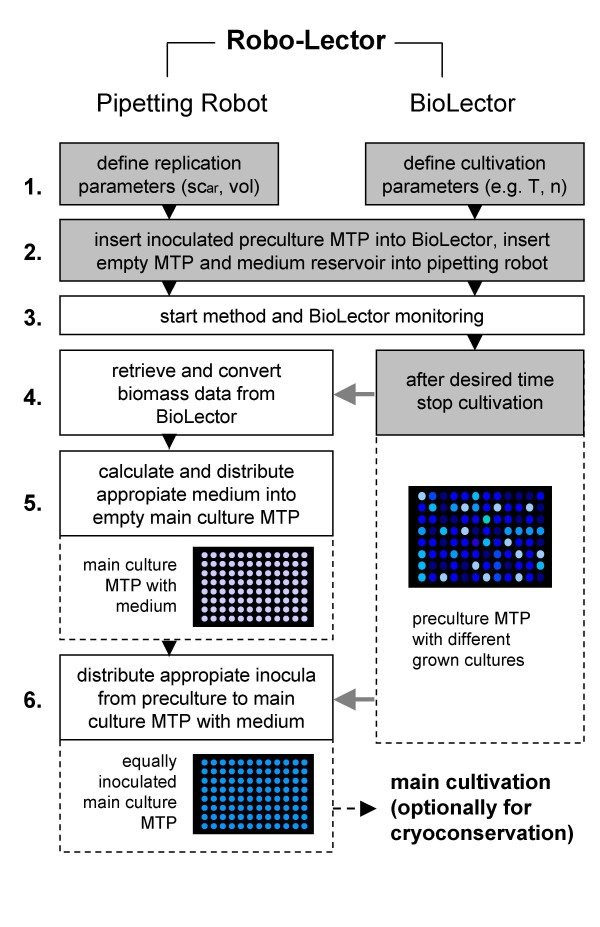
**Principle of method *biomass-specific replication***. Simplified process flow chart; shaded boxes indicate necessary user input; gray arrows indicate interaction of pipetting robot and BioLector; MTP: microtiter plate; parameters sc_ar_: scattered light after replication, vol: total volume of wells after replication, T: cultivation temperature, n: shaking frequency; more details are described in the Materials and Methods section.

The principle of this method is to transfer (replicate) cultures from one microtiter plate (preculture) to another one (main culture) taking into account their specific biomass concentrations. This is achieved by permanently monitoring the biomass of the preculture plate with the BioLector and subsequent mixing of fresh medium in a main culture plate with an appropiate amount of inoculum from this preculture. This concept is shown as a simplified flowchart in Figure 4. First, again the user has to specify the cultivation conditions of the BioLector (step 1.). Besides that the parameters for the replication step are defined. This is the target scattered light intensity (sc_ar_) at which all the cultures of a 96 well microtiter plate (main culture) are equally inoculated after the replication step. Another parameter is the total liquid volume of the wells (vol), in which the equally inoculated main cultures are prepared (200 μL in this study).

After setting above parameters for the BioLector and the replication step, an inoculated preculture microtiter plate is placed in the BioLector (step 2.). This plate is prepared as follows. To simulate different growth kinetics during a cultivation, a cryoculture plate (stored at -80°C, containing 150 g/L glycerol) with different biomass concentrations (six different concentrations in triplicate) is thawed, mixed and 10 μL from each well are transferred to the corresponding wells of the preculture plate with 190 μL of MDG medium (giving initial optical densities of 0.2, 0.1, 0.05, 0.025, 0.0125, 0.005). Besides this preculture plate a sterile empty microtiter plate and a reservoir (Nerbe Plus, Winsen, Germany) with MDG-medium is placed inside the pipetting robot. Subsequently the method and the BioLector monitoring is started (step 3.). After a desired time (8.2 h in this study) the cultivation is stopped manually in this study (although this step can also be automized) and the replication is started (step 4.). The pipetting robot connects to the BioLector and retrieves the scattered light data from the cultures of the monitored plate. These data are converted into a specific file for further processing in the method program. Based on the biomass concentration of all cultures it is calculated how much medium and how much inoculum from the preculture is needed to achieve a uniformly inoculated main culture microtiter plate (step 5.). Then, the liquid handling robot pipets the appropiate volume from the medium reservoir into each well of the empty main culture plate. Afterwards the appropiate inoculum volume from the preculture plate is distributed to the main culture plate (step 6.), resulting in an equally inoculated culture plate with the predefined sc_ar _(20 a.u., OD 0.9). This microtiter plate is then used for the main cultivation, which is again monitored with the BioLector.

Another possibility would be to use the equally inoculated culture plate for cryoconservation. Therefore, a portion of a sterile glycerol stock solution could be added to each well by the pipetting robot and afterwards the plate would be frozen.

## Results and Discussion

### Induction profiling

**Figure 5:**
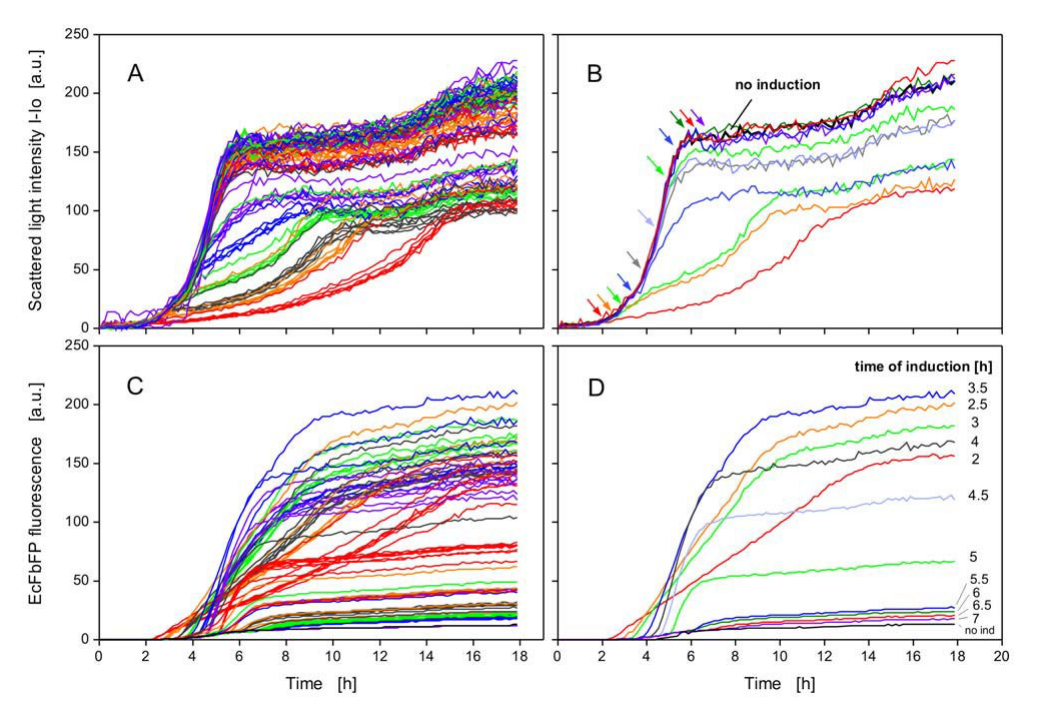
**Influence of induction time and inducer concentration on EcFbFP production of *E.coli *BL21(DE3) pRhotHi-2-EcFbFP**. 96 Induction experiments were conducted according to the profile shown in Figure 2B. Scattered light intensities of all cultures (A) and cultures induced with 0.05 mM IPTG at various points of time, as indicated by arrows (B). EcFbFP production of all cultures (C) and cultures induced with 0.05 mM IPTG (D). In Figure 5B and 5D also a reference cultivation (no induction) is shown; BioLector conditions: 190 μL MDG mineral medium, 3 mm shaking diameter, 950 rpm shaking frequency, 37°C.

In this study the new Robo-Lector platform combining automated liquid-handling and cultivation in monitored microtiter plates (Figure 1) was used to study the expression of a fluorescent model protein from the strain *E. coli *BL21(DE3) pRhotHi-2-EcFbFP. The automated method designated 'induction profiling' was used as shown in Figure 2 and resulted in 96 growth and product formation kinetics (Figure 5). The growth of all cultures, being induced at various times with various IPTG concentrations during the batch cultivation, varied as well as the product formation (Figure 5A and 5C). To simplify the evaluation of data shown in Figure 5A and 5C, only the curves of the 0.05 mM IPTG induction experiments are depicted in Figure 5B and 5D. The uninduced culture showed a typical sigmoid batch cultivation curve (Figure 5B, black line) with a stationary phase starting at 6 h at a scattered light of 160 a.u. (OD 7.1) and no significant expression of the fluorescence protein (20 a.u., Figure 5D). When protein expression was induced at the very beginning of the batch culture (2 h, red line), the growth rate decreased markedly, whereas the product signal rose. The EcFbFP expression continuously increased with a constant rate until the cells entered the stationary phase after 14 h. A later induction of the cells (2.5 to 4 h) led to a sharper increase and greater values of the EcFbFP expression due to the higher biomass at the point of induction. When induction occured after 4 h, the EcFbFP expression sharply decreased (Figure 5D), resulting from the transition from the late exponential to the stationary phase and hence a lack of nutrients and metabolic activity of the cells. Therefore, the EcFbFP and also the growth kinetics of these late-induced cultures showed a similar curve shape as the uninduced cultures (Figure 5B and 5D).

Unlike the non-induced cultures, the early induced cultures grew significantly slower. This phenomenon can be attributed to the so-called 'metabolic burden' through recombinant protein expression [33,34]. Metabolic burden is still poorly understood [35], because there are no appropiate methods to monitor it on-line [36]. Recent research focuses on in-depth analysis of the molecular physiological reactions in host cells during protein expression, e.g. through the use of methods such as DNA microarrays or 2D-electrophoresis [37]. Unfortunately, such methods are complex and quite time-consuming. The approach to fuse green fluorescent protein (GFP) with stress-sensitive promoters and to measure fluorescence is an easier and promising way to achieve on-line-monitoring of the metabolic burden during protein expression in bioreactors [37]. Until now such studies have been mainly conducted with larger bioreactor configurations that can be more easily equipped with fluorescence probes than most MBRs. However, with the Robo-Lector platform presented here, it is now possible to provide on-line fluorescence measurements in parallel experiments and to automate high-throughput cultivations. This platform thus provides an easy and efficient tool to systematically study and possibly quantify metabolic burden.

**Figure 6:**
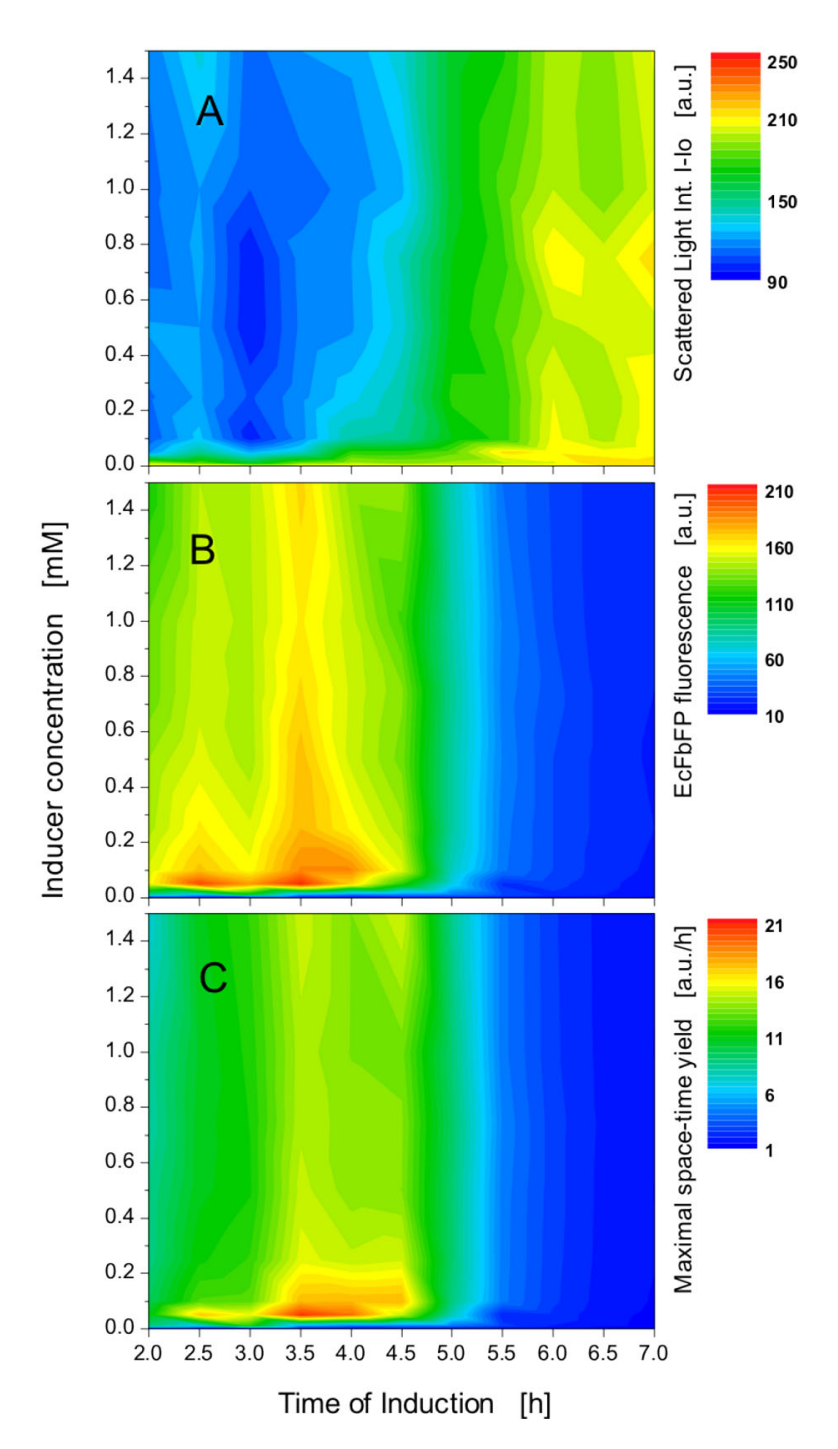
**Induction profiles of *E.coli *BL21(DE3) pRhotHi-2-EcFbFP**. (A) Scattered light intensity and (B) EcFbFP fluorescence; data taken from 96 expression experiments of Figure 5A and 5C at the end of the cultivation (18 h). (C) Maximal space-time yield at various inducer concentrations and induction points; the maximal space-time yield was calculated from the curves of Figure 5C by dividing the EcFbFP fluorescence by the corresponding time.

By systematically varying the induction point and inducer concentration over a wide range in parallel experiments, it is possible to obtain a so-called 'induction profile' of the specific strain tested. Such a profile is presented in Figure 6, where data from the experiment of Figure 5 (at endpoint of cultivation) were plotted as contour plots. These color-coded profiles can also be calculated at any time of the process because of the extensive data provided by the BioLector. For example, in this study the biomass and fluorescence in each of the 96 wells has been detected every 5 min, resulting in over 20,000 data points. The contour plot of the scattered light signal at the end of the experiment reveals that biomass concentration peaks when induction occurs after 4.5 h, independent of the inducer concentration (Figure 6A; green to yellow region). Only the uninduced cultures (inducer 0.0 mM) also show high biomass concentrations. Even though this can also be seen in Figure 5A, the contour plot as shown in Figure 6A depicts this bundle of information much easier and in a concise way.

The fluorescence of the EcFbFP is greatest (> 200 a.u.; red region) at an inducer concentration of only 0.05 to 0.1 mM and an induction time at 2.5 to 3.5 h (Figure 6B). High to moderate EcFbFP production (160 to 200 a.u.; yellow to red region) is also reached at an induction time of 3 to 4 h. Interestingly, at this time the protein expression is much less dependent on the inducer concentration above 0.2 mM IPTG. Miao and Kompala, using a similar host/vector system, have also found that protein expression stagnates at inducer concentrations greater than 0.2 mM [10]. However, this observation results from different possible factors that are not the focus of this paper. When induction takes place after 4 h, there is a sharp decrease in EcFbFP production (green to blue region), as already discussed for Figure 5D. Upon comparing Figure 6A with 6B, it becomes obvious, the more protein expressed, the lower the biomass growth is. This effect can also be explained by the metabolic burden which the recombinant protein expression exhibits on the host bacteria. Figure 5D shows that when induction occurs after 2 h, one obtains the same product quantity as after induction at 4 h. Only the product formation rate is different. Since the culture induced after 2 h reaches its maximum EcFbFP fluorescence approximately 6 h later than the 4 h-induced culture, it makes sense to basically induce cultures at 4 h to save time. This aspect is not visible in Figure 6B. Hence, the maximum EcFbFP produced per time (maximum space-time yield) would be a better indicator to distinguish between different induction experiments. Furthermore, maximizing the space-time yield is the principal goal of biological production processes [13]. Thus, the space-time yield has been calculated from the data of Figure 5C by dividing the EcFbFP fluorescence (a.u.) by the corresponding time (h). The maxima of the resulting space-time yield curves are depicted in Figure 6C. This space-time yield calculation results in a distinct region with the highest EcFbFP production per time (yellow to red) at inducer concentrations of 0.05 to 0.2 mM added to the culture after 3.5 to 4.5 h. Comparing this with Figure 6B clearly shows that inducing these specific cultures earlier than 3.5 h or at higher IPTG concentrations makes no sense in respect to fast and high product formation. It should be emphasized that different recombinant strains may behave completely different. The induction profiling method presented here allows to automatically conduct and monitor up to 96 induction experiments in just one run (e.g. over night), thereby allowing the researcher to attain a fast, easy and profound understanding of the given expression system, especially when fluorescent marker proteins are used.

### Biomass-specific induction

**Figure 7:**
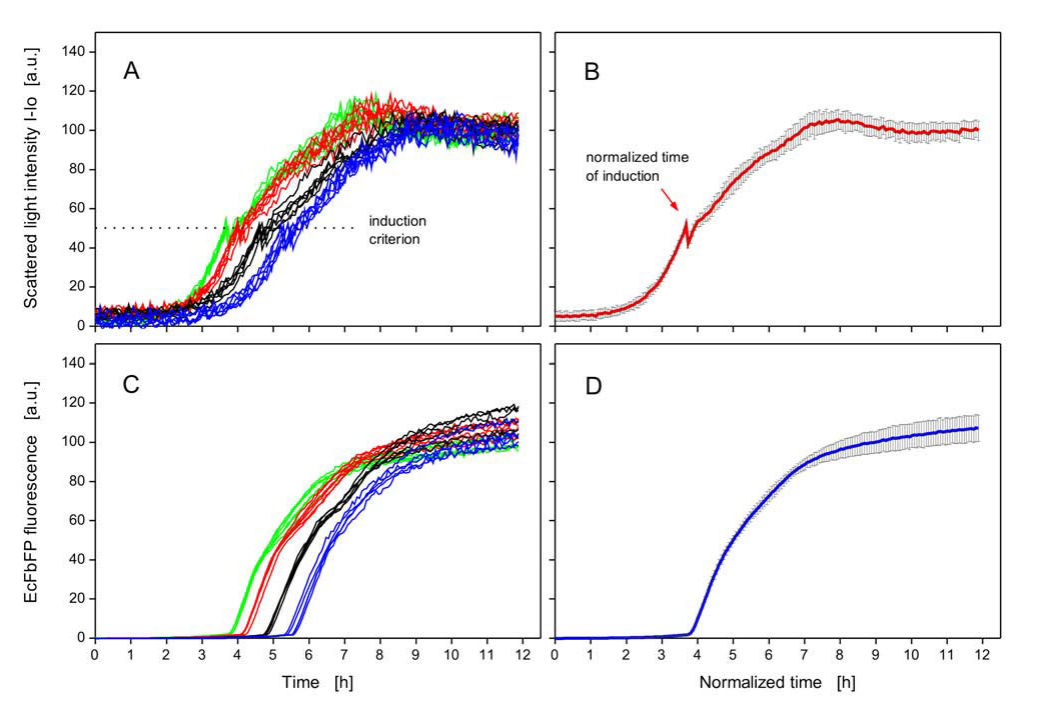
**Effects of *biomass-specific induction *on *E.coli *BL21(DE3) pRhotHi-2-EcFbFP**. (A) Growth of different inoculated cultures, induced at an induction criterion of 50 a.u. with 0.1 mM IPTG; cultures from left to right (in sixfold): optical density at start = 0.16 (green curves), 0.11 (red curves), 0.05 (black curves), 0.03 (blue curves). (B) Mean and standard deviation of normalized growth of 24 cultures from Figure 7A; the growth kinetics were normalized to have the same induction time as the first induced culture. (C) EcFbFP production of all induced cultures. (D) Mean and standard deviation of normalized product formation of 24 cultures from Figure 7C. BioLector conditions: 190 μL MDG mineral medium, 3 mm shaking diameter, 950 rpm shaking frequency, 37°C.

As demonstrated in Figure 5, induction at different growth phases leads to great variations in product formation. Thus, it is a large problem to simultaneously induce cultures which show different growth kinetics with the conventional method (induce all cultures of a microtiter plate at the same time). Since the BioLector permanently monitors growth in microtiter plates, it is possible to trigger actions of the liquid-handling robot in response to the scattered light data. This allows one to conduct a biomass-specific induction of different cultures (that means at different appropiate times according to the requirement of the individual culture) in a completely automated way. Results of this method (Figure 3) are depicted in Figure 7.

To simulate different growth kinetics, some wells of the microtiter plate were inoculated with different amounts of preculture. These growth differences can be seen in Figure 7A. When the first cultures reached the induction criterion of 50 a.u. (OD 2.2) for the scattered light signal, the Robo-Lector automatically induced the corresponding wells of the microtiter plate with IPTG. In Figure 7A, the scattered light curves show a short drop in the signal intensity after induction, caused by dilution of the culture through the added inducer solution. After the automated induction, the growth is immediately slowed down (Figure 7A, also visible in Figure 5A) because of the starting EcFbFP production, thereby leading to an increase in the fluorescence signal (Figure 7C). As the growth curves in Figure 7A and the product curves in Figure 7C of the wells treated in the same way (curves with same colors) are similar, this demonstrates that the automated induction took place at a comparable growth phase. This fact is also supported by normalizing all culture curves to have the same induction time as the first induced culture and by calculating the mean and standard deviation of these curves (Figure 7B and 7D). The EcFbFP fluorescence after 12 h ranges from 100 to 120 a.u. (Figure 7C) with a mean fluorescence of 107 ± 7 a.u. (Figure 7D), representing a relative standard deviation of only ± 7% (the accuracy of the measurement system is ± 5%, according to the manufacturer of the BioLector). Upon comparing Figure 7C with Figure 5C, this differences in product yield is reasonably smaller because of the induction at a similar growth phase. The demonstrated data here show the feasibility of this concept and can be important for applications where an action (e.g. addition of liquids, drawing samples) has to be triggered depending on the growth phase of different cultures.

### Biomass-specific replication

**Figure 8:**
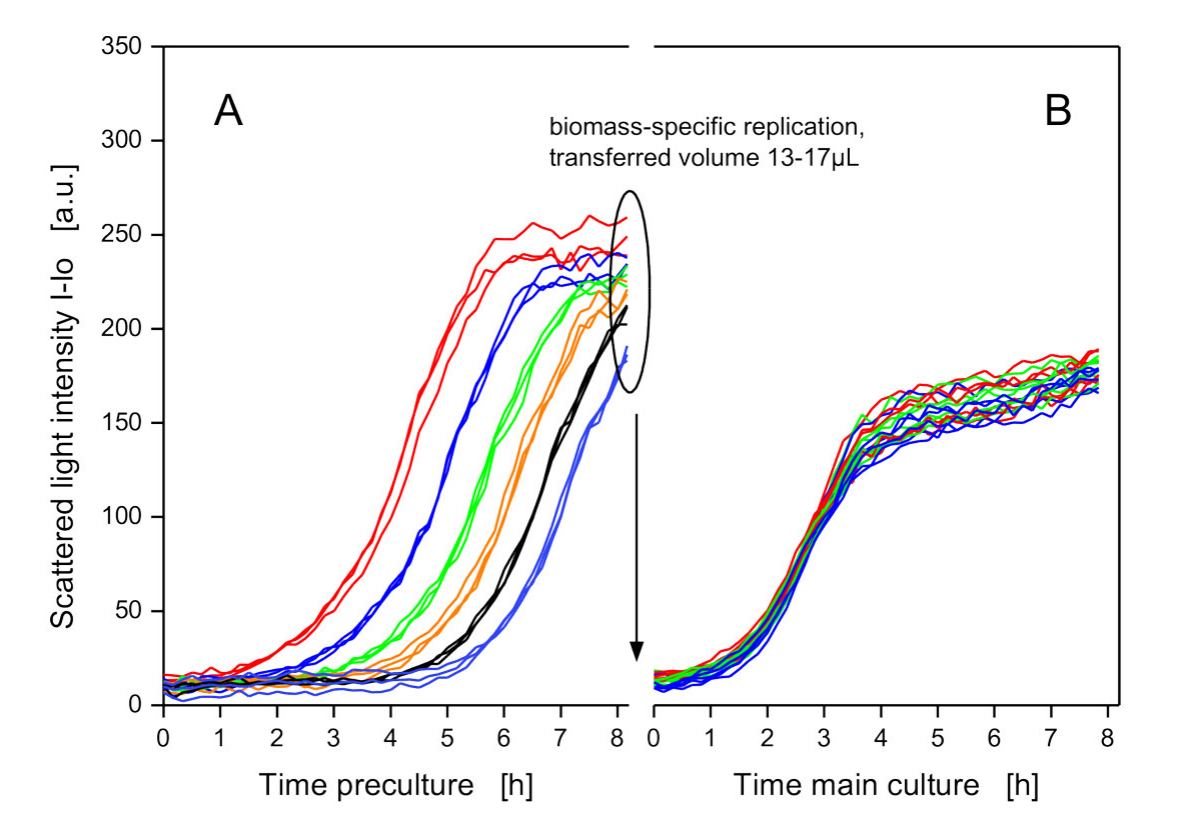
**Results of the method *biomass-specific replication***. (A) Growth of precultures with different initial biomass concentrations, from left to right (in triplicate): optical density at start 0.2, 0.1, 0.05, 0.025, 0.0125, 0.005; after 8.2 h the first cultivation was stopped and the automated method for replication (see also Figure 4) started. (B) Main culture: growth of 18 replicated cultures; the relative standard deviation of the scattered light after 7.8 h is ± 4%. BioLector conditions: 200 μL MDG mineral medium, 3 mm shaking diameter, 950 rpm shaking frequency, 37°C, strain *E.coli *BL21(DE3) pRhotHi-2-EcFbFP.

To simulate different growth kinetics in high-throughput cultivations, a preculture microtiter plate with different initial biomass concentrations was used. The resulting growth differences can be seen in Figure 8A. After 8.2 h this cultivation was stopped. At that moment, the different cultures had scattered light intensities of 240 to 300 a.u. and were at different phases of the batch cultivation. Some of the cultures were already in the stationary phase, whereas others were still in the late-exponential growth phase (Figure 8A). At this time, the biomass-specific replication was conducted (Figure 4). Fresh medium was distributed to a new microtiter plate and appropiate amounts of the cultures from the preculture plate (13 to 17 μL) were transferred to the corresponding wells of the main culture plate to give a final volume of 200 μL per well with a scattered light intensity of approximately 20 a.u. (sc_ar_). The subsequent main cultivation showed that all 18 cultures had very similar growth kinetics after the biomass-specific replication (Figure 8B) with a relative standard deviation of ± 4% regarding scattered light (after 7.8 h), demonstrating that the method can provide equal starting conditions and hence growth kinetics in the main culture.

This method can be used to systematically study multiple cultivation steps, such as preculture-main culture cascades and influencing factors of the inoculum on a main culture (e.g. lag-time, volume ratio of the preculture to fresh medium). If the differences in biomass concentration in different preculture wells are too large to be equalized in one single replication step, it is also possible to include another preculture before the main culture an to conduct the biomass-specific replication twice.

## Conclusion

In industry and academic research, there is an increasing demand for flexible automated microfermentation platforms with advanced sensing technologies. The Robo-Lector, a new platform constisting of a BioLector and a liquid-handling robot, has been sucessfully built and tested. Unlike other MBR systems, the BioLector generates extensive kinetic data in high-throughput cultivations concerning biomass and fluorescence protein formation. Based on the non-invasive on-line-monitoring signal for microbial growth, actions of a liquid-handling robot can easily be triggered and controlled. This interaction between the robot and the BioLector combines high-content data generation with systematic high-throughput experimentation in an automated fashion, offering new possibilities to study biological production systems.

The 'induction profiling' and 'biomass-specific induction' methods presented here allow one to study recombinant protein expression in detail and optimize expression. This, in turn, leads to a fast and profound comprehension of host/vector systems and the metabolic burden phenomenon. The method 'biomass-specific replication' enables to generate main cultures with equal biomass concentrations from different growing precultures. Additionally, the presented method could be useful for establishing standardized cryocultures and to study the optimal time for freezing the cells in respect to the lag-time or the vialbility of the cryocultures. The novel automated methods presented here can aid in modelling recombinant protein expression, systems biology research and in particular bioprocess development and optimization. In these fields, the versatile platform can accelerate and intensify research and development.

As the Robo-Lector has a simple but efficient design (microtiter plate format; use of standard liquid-handling robot and hence widespread automation possibilities), it can be easily combined with established techniques for small scale downstream processing [38-41] and analytical assays. This fits the current trend for small-scale process integration in just one automated platform [4]. Liquid-handling workstations permit high-throughput sampling and addition of liquids to microtiter plates so that for example intermittent fed-batch processes with the Robo-Lector can be realized. Furthermore, as liquid-handling workstations allow fast and accurate pipetting, many different media compositions can be generated in the Robo-Lector. This enables one to combine media preparation with subsequent parallel cultivations to optimize media in an automated fashion, e.g. with the help of DoE (design of experiments) [42] or genetic algorithms [43,44]. Methods implementing fed-batch and automated media preparation have already been established for the presented platform (data not shown).

Ultimately, the Robo-Lector can contribute to the envisioned paradigm shift in bioprocess development [4]. This entails switching from rather empirical process development (low throughput experimentation with no or unsophisticated monitoring) to high-throughput experimentation with very sophisticated yet simple monitoring to generate deeper knowledge of biological production systems.

## Competing interests

The authors declare that they have no competing interests.

## Authors' contributions

RH made the conceptual design of the presented platform, designed the experimental setup and methods and prepared the manuscript. DR automated and tested methods, performed cultivation experiments. TH programed the pipetting robot. AKH performed cultivation experiments. FK designed the custom-made BioLector. LW provided command codes for controlling the BioLector. CM designed the custom-made BioLector. JB initiated the project, assisted with conception, data interpretation and manuscript preparation. All authors read and approved the final manuscript.
